# Targeting AMP-activated protein kinase in sepsis

**DOI:** 10.3389/fendo.2024.1452993

**Published:** 2024-10-14

**Authors:** Tetsuya Yumoto, Craig M. Coopersmith

**Affiliations:** ^1^ Department of Surgery and Emory Critical Care Center, Emory University School of Medicine, Atlanta, GA, United States; ^2^ Department of Emergency, Critical Care and Disaster Medicine, Faculty of Medicine, Dentistry and Pharmaceutical Sciences, Okayama University, Okayama, Japan

**Keywords:** AMP activated kinase, metformin, AICAR, sepsis, treatment

## Abstract

Sepsis is a global health challenge marked by limited clinical options and high mortality rates. AMP-activated protein kinase (AMPK) is a cellular energy sensor that mediates multiple crucial metabolic pathways that may be an attractive therapeutic target in sepsis. Pre-clinical experimental studies have demonstrated that pharmacological activation of AMPK can offer multiple potential benefits during sepsis, including anti-inflammatory effects, induction of autophagy, promotion of mitochondrial biogenesis, enhanced phagocytosis, antimicrobial properties, and regulation of tight junction assembly. This review aims to discuss the existing evidence supporting the therapeutic potential of AMPK activation in sepsis management.

## Introduction

Sepsis represents a significant global health challenge, impacting nearly 50 million people and accounting for approximately 20% of all deaths worldwide (1). Despite advances in understanding its pathophysiology, septic shock often results in mortalities of 30% or higher. Management of sepsis is generally supportive. While fluids and antibiotics improve outcome, no pharmacologic therapy has been shown to effectively target the dysregulated host response seen in sepsis (2, 3). While numerous preclinical studies have shown promise in improving outcomes based upon sound mechanistic insights, effective translation of specific treatments for sepsis into clinical practice at the bedside has proven to be an elusive goal (4). Consequently, there is a clear need to develop targeted and effective therapeutics to augment current strategies in sepsis management.

AMP-activated protein kinase (AMPK) is a central integrator of cellular energy and metabolic homeostasis (5). Once activated, AMPK participates in triggering downstream effector proteins involved in a range of biological responses, from glucose metabolism and lipid oxidation to autophagy (6, 7). Although AMPK activation is best known for its role in long-term treatment of diabetes through drugs such as metformin, growing evidence suggests that AMPK activation can exert anti-inflammatory effects through several mechanisms. As such, AMPK is increasingly viewed as a potential therapeutic target for a number of human conditions, including cardiovascular diseases, ischemia-reperfusion injuries, and various inflammatory diseases. Numerous pharmacological agents have been identified that can activate AMPK, ranging from widely used medications to innovative substrates to natural compounds.

In critically ill patients, multiple meta-analyses have demonstrated an association between preadmission use of metformin, an indirect AMPK activator, and lower mortality in adult septic patients who have diabetes mellitus (8, 9). Further, a propensity score matched cohort of 2,691 septic ICU patients with type 2 diabetes mellitus demonstrated decreased 90 day mortality, reduced severe kidney injury and increased renal recovery in patients exposed to metformin during their hospitalization (10). Additionally, metformin exposure during *Staphylococcus aureus* bacteremia (regardless of prior use) was shown to be an independent predictor of survival in a study of 452 patients with diabetes (11). Understanding the limitations of associative studies and that causation should not be inferred from these studies, activation of AMPK may represent a therapeutic strategy for the treatment of sepsis that is worthy of further study. In fact, a protocol has recently been published for a planned randomized clinical trial examining the safety and feasibility of metformin as a treatment for sepsis-associated acute kidney injury (12). While metformin is well known as a treatment for diabetes mellitus, AMPK activation has numerous effects above and beyond this, and the full spectrum of effects of AMPK activation during sepsis remains to be fully elucidated. This review explores the current understanding of the role of AMPK in sepsis, emphasizing the therapeutic implications of AMPK activators.

### Overview of AMPK

AMPK functions as an energy sensor that is activated in response to cellular energy depletion. Activation is triggered by increased AMP/ATP or ADP/ATP ratios during conditions such as starvation, hypoxia, ischemic stress, or exercise. Structurally, AMPK is a heterotrimeric complex comprised of three subunits: the catalytic α-subunit, the scaffolding β-subunit, and the regulatory γ-subunit. There are two isoforms of the α and β subunits, while the γ-subunit has three isoforms (13, 14). The α1, β1, and γ1 subunits are ubiquitously expressed, whereas other combinations display tissue-specific expression. For instance, the α2 and β2 subunits are predominantly found in heart and skeletal muscle (15). Primary physiological AMPK activation is achieved by phosphorylation at Thr172 of the AMPK α-subunit predominantly by upstream kinases including liver kinase B1 (LKB1) and calcium/calmodulin-dependent protein kinase kinase-beta (CaMKKβ) (5, 14). Activation of the LKB1/AMPK signaling pathway reduces lung vascular permeability and the systemic inflammatory response following lipopolysaccharide (LPS) treatment (16). Macrophage LKB1 also helps control local *Klebsiella pneumoniae* growth during pneumonia by maintaining adequate alveolar macrophages in the lung (17). In contrast, CaMKKβ activates AMPK via an increase in intracellular Ca^2+^ concentration, independent of the AMP/ATP or ADP/ATP ratios. The CaMKKβ/AMPK pathway is pivotal in providing protection against LPS-induced neuroinflammation and cerebral ischemia/reperfusion injury (18, 19). Activation of AMPK then influences a number of downstream pathways related to carbohydrate, amino acid, and lipid metabolism, as well as mitochondrial function, autophagy, and cell growth (14, 16).

### AMPK activators

AMPK can be activated directly or indirectly by a number of different pharmacological compounds. 5-aminoimidazole-4-carboxamide ribonucleoside (AICAR) is commonly utilized as an experimental direct AMPK activator. AICAR is transformed intracellularly into AICAR monophosphate, which acts as an AMP mimetic, consequently activating AMPK. However, it is worth noting that AICAR also activates other AMP-regulated enzymes, such as fructose-1,6-bisphosphatase through which it could play a role in regulating glycolysis and gluconeogenesis unrelated to AMPK (13). Over the past decade, more specific direct small molecule AMPK activators have been developed. For instance, A-769662 specifically activates AMPK by directly binding to the AMPK β-subunit, resulting in allosteric activation. In contrast, metformin, a frequently prescribed medication for type 2 diabetes mellitus, along with other biguanides, indirectly activates AMPK by inhibiting the mitochondrial respiratory chain complex I, leading to an increase in AMP levels. Additionally, IM156 is a novel biguanide more potent than metformin in activating AMPK. Several natural products derived from plants, such as berberine from traditional Chinese medicine and the polyphenol resveratrol, have also been identified as AMPK activators (20, 21).

### Kinetics of AMPK activation during sepsis

AMPK activation changes during sepsis differ depending on both tissue and species. AMPK activation is increased at 12 hours and declines at 24 hours following CLP compared with sham control in mouse liver (22). In contrast, AMPK activation is elevated at 6 hours in rat heart and remains elevated at 24 and 72 hours following CLP compared with sham control (23). A trend towards lower AMPK activation is observed in mouse lung 24 hours after CLP (24) while AMPK activation trends higher by 6 hours in mouse kidney after CLP, before returning to baseline (25). AMPK activation in diaphragm and tibialis anterior is also increased 48 and 96 hours after CLP in mice (26). In contrast, human biopsy data demonstrated that in vastus lateral muscle, AMPK activity is reduced in critically ill ICU patients (not restricted to sepsis), with an interquartile range of 4 to 6 days following admission (27).

### Potential beneficial effects of AMPK activation in treating sepsis

**Figure 1:**
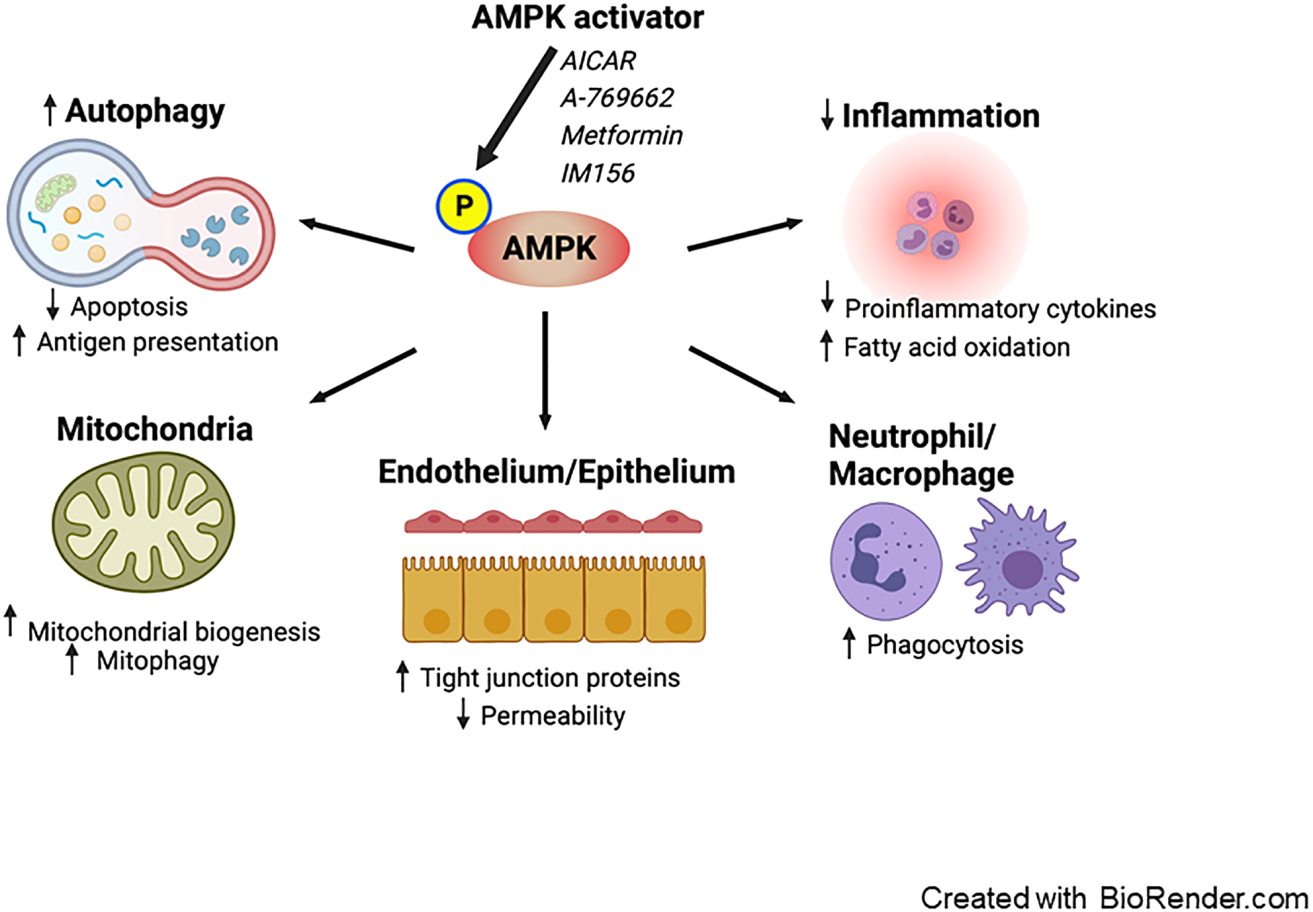
Proposed effects of AMPK activation on sepsis. Pharmacological activation of AMPK enhances autophagy, leading to a decrease in apoptosis and an increase in antigen presentation. AMPK activation promotes mitochondrial biogenesis and mitophagy. It exerts anti-inflammatory effects by reducing the production of pro-inflammatory cytokines and facilitating fatty acid oxidation. Furthermore, AMPK boosts the phagocytic activity of neutrophils and macrophages. Additionally, AMPK reduces vascular and intestinal permeability by restoring the integrity of tight junctions in the endothelium and epithelium. AMPK; AMP-activated protein kinase.

Experimental studies (described in further detail below) have demonstrated that metformin can potentially mitigate sepsis-induced organ failure via AMPK activation (28) via a number of physiological benefits (**Figure 1**).

### Anti-inflammatory effect

Sepsis is characterized as having dysregulated inflammation. While the balance between inflammation and immunosuppression is complex, modulating excessive inflammation – especially in the early stages of sepsis – and the resulting organ damage stands out as a potential therapeutic target (29). The role of AMPK in inflammation regulation has been extensively characterized. AMPK activation indirectly suppresses nuclear factor-κB, a pivotal transcriptional factor that induces the inflammatory response, through various mediators including SIRT1, FOXO, and PGC1α (30). Diminished AMPK activity in macrophages leads to a marked increase in the expression of pro-inflammatory cytokines IL-6 and TNF following LPS treatment with a simultaneous downregulation of the anti-inflammatory cytokine IL-10 (31). In contrast, AMPK activation causes a decrease in LPS-induced IL-6 and TNF production, coupled with a surge in IL-10 production. In addition, AMPK exhibits anti-inflammatory properties by modulating lipid metabolism. It is recognized that sepsis impacts fatty acid metabolism, with systemic inflammation and lipotoxicity stemming from reduced fatty acid oxidation and an accumulation of free fatty acids (32, 33). AMPK aids in enhancing fatty acid oxidation and mitigating excessive lipid accumulation via the phosphorylation of acetyl-CoA carboxylase (5).

### Induction of autophagy

Autophagy is a dynamic process through which intracellular materials are degraded and subsequently recycled in lysosomes. In mouse cecal ligation and puncture (CLP), a model of polymicrobial intraabdominal sepsis, autophagy is accelerated 6 hours after the induction of sepsis, but this is followed by a decline in autophagy in the liver, heart, and spleen (34).

Some preclinical studies have indicated that autophagy offers protective effects against sepsis in multiple vital organs including lung, heart, kidneys and brain although it may be potentially harmful in skeletal muscle (35). The beneficial aspects of autophagy can be attributed, in part, to the preservation of mitochondrial integrity, the prevention of apoptosis, and the enhancement of MHC II antigen presentation (36). Interestingly, suppressing autophagy in the liver heightens mortality rates after experimental sepsis, primarily due to the induction of apoptosis and mitochondrial damage (37). Thus, autophagy modulation might be a promising therapeutic target for sepsis treatment. The Unc-51-like autophagy-activating kinase 1 (ULK1) protein kinase is pivotal in the initiation of autophagy. AMPK acts directly as an upstream signal facilitating ULK1 activation. Moreover, AMPK suppresses the mechanistic target of rapamycin (mTOR), another main regulator that exerts negative control over autophagy (38).

### Mitochondrial homeostasis

Mitochondria are responsible for numerous physiological processes, including ATP generation through oxidative phosphorylation, reactive oxygen species (ROS) production, calcium homeostasis, and the initiation of apoptosis. Mitochondrial homeostasis hinges on two opposing yet harmoniously coordinated processes: mitochondrial biogenesis and mitochondrial selective autophagy (often referred to as mitophagy) (39). Mitochondrial biogenesis is the process through which mitochondria adapt by increasing both in number and size, ensuring their robust heath and functionality. In contrast, mitophagy is a form of specialized autophagy targeting the selective degradation of damaged mitochondria via lysosomes. Beyond general autophagy, ULK1 is believed to be integral to mitophagy under various conditions (6).

Sepsis frequently leads to mitochondrial dysfunction, which has been mechanistically linked to poor patient outcomes (40, 41). This dysfunction is mainly marked by disturbance in electron transport chain function, causing excessive ROS production. The surge in ROS is further exacerbated by pro-inflammatory cytokines, such as IL-1, IL-6, and TNF. This cascade eventually triggers apoptosis, induced by the release of cytochrome c and other pro-apoptotic proteins (42, 43). Emerging evidence highlights the protective roles of both mitochondrial biogenesis and mitophagy during sepsis. In response to mitochondrial dysfunction, AMPK activation serves as a key compensatory mechanism by stimulating both mitochondrial biogenesis and mitophagy, restoring function and reducing damage (41, 44–46). Notably A-769662, a potent AMPK activator, bolsters mitochondrial biogenesis, as evidenced by an increase in nuclear PGC-1α activity within the lungs (47). Additionally, pre-treatment with lycorine, a benzyl isoquinoline alkaloid, decreases mortality and mitigates cardiac injury following CLP, by activating AMPK and suppressing ROS production and oxidative stress (48). In contrast, inhibiting mitophagy in macrophages leads to enhanced survival following sepsis. This phenomenon is attributed to ROS production in mitochondria, facilitating bacterial clearance and host defense (49).

### Role of AMPK in immune cells

AMPK has been demonstrated to play a crucial role in the immune response, particularly within myeloid and T cells. Activating AMPK with either metformin or AICAR leads to reduced bacterial loads in peritoneal lavage following peritonitis-induced sepsis, associated with the amplification of neutrophil chemotaxis, phagocytosis, and bacterial neutralization (50). Additionally, metformin-induced AMPK activation inhibits the proliferation of *Listeria pneumophilia* within macrophages through enhanced mitochondrial ROS production (51). Moreover, AMPK is an important regulator of high mobility group box 1 (a consequential late mediator in lethal sepsis) release within stimulated macrophages and monocytes (52). AMPK is also crucial for T cell-mediated immunity as mice with a T cell-specific deletion of AMPKα1 have reduced counts of CD4^+^ and CD8^+^ T cells within bronchoalveolar lavage and lung tissues following pulmonary infections caused by either Influenza A or *Listeria monocytogenes* (53). While viral load in the lung remains consistent in mice with T cell-specific AMPKα1 deletion following Influenza A, knockout mice exhibit elevated bacterial loads within the liver (with a trend towards an increase in the spleen) following *Listeria monocytogenes.*

### Antimicrobial effect

AMPK plays a complex role in infectious diseases, especially viral infections, with its impact varying depending on the specific pathogen. AMPK activation also plays a key role in defending the host against *Mycobacterium tuberculosis*. This bacterium bypasses the host’s autophagic defense system in part by activating the mTOR pathway. AMPK therefore has been highlighted as a potential therapeutic target in the fight against mycobacterium infection (54). Within severe acute respiratory syndrome coronavirus 2 (SARS-CoV-2), AMPK activation leads to the phosphorylation and stabilization of angiotensin-converting enzyme 2 (ACE2) (55). While ACE2 facilitates SARS-CoV-2 entry into host cells, it also offers protection against lung injury by reducing inflammation, fibrosis, and pulmonary arterial hypertension (56). Metformin was extensively studied for its potential anti-COVID-19 effects. Based on lack of efficacy in the TOGETHER and COVID-OUT trials, metformin is not recommended for treatment of patients with COVID-19 in international guidelines; however, interest remains in identifying subsets of patients in whom it may be beneficial (57–60). IM156, a novel biguanide more potent than metformin in activating AMPK, offers protection following CLP by enhancing bacterial clearance, regulating cytokine release, and preventing lymphocyte apoptosis (61). IM156 also inhibits ROS generation and formation of neutrophil extracellular traps in response to LPS, so the mechanism underlying its survival benefit following sepsis is likely to be multifactorial.

Cytomegalovirus (CMV) reactivation is frequently seen in critically ill patients without primary immunodeficiency diseases (62). CMV relies on AMPK for glycolytic activation during its replication and suppressing AMPK activity can hinder CMV replication (63). Additionally, the effects of AMPK activation on bacterial infections vary based on the specific host cells infected and the nature of the pathogen. A comprehensive review of the impact of host AMPK activation on various microbial species has been conducted and can be found elsewhere (64).

### Regulation of epithelial/endothelial tight junction assembly

AMPK activation exerts beneficial impacts on tight junction assembly. For instance, AMPK activation using metformin and AICAR has been shown to counteract airway epithelial barrier disruption caused by *Pseudomonas aeruginosa*, a significant microorganism linked to hospital-acquired infections in critically ill patients (65).

The intestinal epithelium serves as a dynamic protective barrier, separating the host from its external environment. This barrier’s integrity is regulated by both tight junction-dependent and independent mechanisms (66, 67). Gut barrier dysfunction characterized by intestinal hyperpermeability is associated with various diseases and systemic inflammation (68). AICAR bolsters ZO-1 formation, leading to a reduction in intestinal paracellular permeability following heat stress in rats (69). In a model of experimental colitis, metformin-induced AMPK activation upregulates the expression of occludin, ZO-1, and claudin-1, mitigating colonic damage (70). Current evidence suggests that sepsis impairs tight junctions in two different pathways (pore and leak), associated with intestinal hyperpermeability and unfavorable outcomes (67, 71). To our knowledge, there have been no studies investigating the impact of AMPK activation on intestinal barrier function in sepsis.

Disruption of endothelial tight junction proteins also worsens microvascular permeability and exacerbates multiple organ dysfunction (72). A number of investigations have centered on the interplay between AMPK activity and endothelial permeability in the context of LPS stimulation. AMPKα1-deficient mice display elevated cardiac vascular permeability, reduced endothelial ZO-1 expression, and myocardial edema post-LPS exposure (73). Conversely, in wild-type mice, these adverse effects are counteracted by AICAR. Additionally, both metformin and AICAR are effective in alleviating LPS-induced pulmonary endothelial permeability in rats (74).

### AMPK activation as a therapeutic target in sepsis

Regardless of the degree of AMPK activation in tissues following sepsis, a large number of pre-clinical studies have demonstrated beneficial effects of AMPK activation following either CLP-induced sepsis or sterile inflammation in LPS-induced endotoxemia. **Table 1** summarizes the effects of AMPK activators or drugs/agents that can positively mediate AMPK pathway in animal studies (22, 23, 25, 47, 61, 74–104). Notably, a single dose of metformin administered 1 hour after CLP improves survival associated with preservation of metabolic fitness (25). Additionally, Dexmedetomidine, a sedative commonly used for critically ill patients, enhances hepatic autophagy through AMPK activation and leads to improved survival compared to vehicle in mice subjected to CLP (22).

**Table 1:** Effects of AMPK activation on CLP-induced sepsis or LPS-induced inflammation.

Model	Species	AMPK activator	Dosage	Survival benefit	Target organ	Mechanisms	References
LPS	Mouse	AICAR	500 mg/kg 4 h before LPS injection	N.A.	Lung	AICAR reduced of TLR4-induced neutrophil activation and proinflammatory responses in lung.	(75)
CLP	Mouse	AICAR	100 mg/kg 24 h before CLP surgery	N.A.	Liver/Kidney	AICAR ameliorated of liver and kidney injury indicated by decrease in proinflammatory response and endothelial activation.	(76)
CLP	Mouse	AICAR	500 mg/kg 1 and 6 h after CLP surgery	Yes	Liver	AICAR promoted mitochondrial biogenesis and autophagy in liver.	(77)
LPS	Rat	AICAR & Metformin	20 mg/kg for AICAR 15 mg/kg for metformin 6 h after LPS injection	Yes	Lung	AMPK activators reduced vascular permeability and edema in lung via AMPK activation.	(74)
CLP	Mouse	AICAR & metformin	500 mg/kg for AICAR 200 mg/kg for metformin 1 h after CLP surgery	Yes	Kidney	AMPK activators improved metabolic fitness including enhancement of fatty acid oxidation and glucose uptake through Sirt3 signaling.	(25)
LPS	Rat	Metformin	100 mg/kg 30 min before LPS injection	N.A.	Heart	Metformin prevented cardiac dysfunction by suppression of TLR4 activity through AMPK activation.	(78)
LPS	Mouse	Metformin	250 mg/kg 16 h before LPS injection	N.A.	Lung/Kidney	Metformin ameliorated endothelial proinflammatory responses via mediating AMPK/HDAC5/KLF2 signaling.	(79)
LPS	Mouse	Metformin	100 mg/kg for a week before LPS injection	N.A.	Heart	By activating AMPK/mTOR) pathway	(80)
LPS	Mouse	A769662	30 mg/kg 30mins before LPS injection	N.A.	Heart	A769662 improved cardiac function and upregulated cardiac autophagy via AMPK/mTOR pathway.	(81)
CLP	Mouse	A769662	10 mg/kg 1 h after CLP surgery	Yes	Lung	A769662 ameliorated lung injury by activating nuclear AMPKα1/α2 and AMPKβ1 subunit and autophagy.	(47)
CLP	Rat	Adiponectin	3 mg/kgA Right after CLP surgery	N.A.	Liver	Adiponectin attenuated liver injury by modulating AMPK/mTOR pathway.	(82)
LPS	Mouse	Artemisinin (antimalarial drug)	30 mg/kg 4 days before LPS injection for 5 consecutive days	N.A.	Brain (hippocampus)	Artemisinin improved LPS-induced cognitive impairments and suppressed proinflammatory cytokines by activating the AMPKα1 pathway.	(83)
LPS	Mouse	β-Hydroxyisovalerylshikonin	2.5 mg/kg 3 consecutive days before LPS injection	Yes	Lung /Macrophages	Via activation of the AMPK/Nrf2 pathway	(84)
CLP/LPS	Mouse	Cilostazol	10 mg/kg 2 h before and 12 h after LPS injection	Yes	Systemic/Lung	Cilostazol reduced circulating HMGB1 by AMPK activation and induction of HO-1 by p38 MAPK.	(85)
CLP	Rat	Cyclosporine A	10 mg/kg Before CLP surgery	N.A.	Heart	Cyclosporine A attenuated cardiac dysfunction via inhibiting calcineurin and activating AMPK/TCC/CPT1 pathway.	(23)
CLP	Mouse	2-deoxy-D-glucose (2-DG, glycolysis inhibitor)	2 g/kg 3 h before CLP surgery	Yes	Kidney	2-DG alleviated kidney injury by enhancing lactate/Sirtuin 3/AMPK-regulated autophagy.	(86, 87)
LPS	Rat	Dexmedetomidine	30 µg/kg 30 min before LPS injection	N.A.	Kidney	Dexmedetomidine enhanced kideny autophagy via the a2-adrenoreceptor/AMPK/mTOR pathway.	(88)
CLP	Mouse	Dexmedetomidine	20 μg/kg 0, 2, and 4 h after CLP surgery	Yes	Liver	Dexmedetomidine promoted autophagy in liver through the SIRT1/AMPK pathway.	(22)
CLP	Mouse	Ginsenoside Rg3 (Rg3, extract of ginseng)	10 or 20 mg/kg 1 h before CLP surgery	Yes	Liver	Rg3 attenuates mitochondrial dysfunction and promoted autophagy in liver via activating the AMPK signal pathway.	(89)
CLP	Mouse	IM156	5, 10, 15 mg/kg post-CLP	Yes	Lung	Several mechanisms including ROS inhibition	(61)
LPS	Rat	Irisin (myokine)	10 μg/g 30 min before LPS injection	N.A.	Lung	Irisin improved alveolar epithelial barrier dysfunction by activating AMPK/SIRT1 pathway.	(90)
LPS	Mouse	Ketamine	20 mg/kg before LPS injection	N.A.	Lung	By inducing autophagy and reduces apoptosis in lung through the AMPK/mTOR pathway	(91)
CLP	Mouse	Maackiain	2.5 mg/kg or 5 mg/kg 12 h before the CLP	Yes	Systemic	Via activating AMPK/Nrf2/HO-1 pathway	(92)
CLP	Rat	Matairesinol	5-20mL/kg 24 h after surgery	N.A.	Brain	By suppressing the MAPK and NF-κB pathways through up-regulating AMPK	(93)
CLP	Mouse	Melatonin	30 mg/kg before CLP	Yes	Heart	Attenuation of inflammation, oxidative stress, and mitochondrial dysfunction	(94)
LPS	Mouse	13-methylberberine (13-MB)	1 mg/kg 1 h before and 12 h after LPS injection	Yes	Macrophages	13-MB reduced HMGB1 release in macrophages by activating AMPK/p38MAPK pathway.	(95)
CLP	Mouse	Molecular hydrogen	2% hydrogen gas 1 and 6 h after CLP surgery for 1h, respectively	N.A.	Brain (hippocampus)	Hydrogen inhalation attenuated sepsis-induced cognitive impairment by suppressing inflammatory response and enhancing autophagy through AMPK activation.	(96)
LPS	Mouse	Nerolidol (naturally occurring sesquiterpene alcohol found in various plants)	10, 30, and 100 μmol/kg 30 min before LPS injection	N.A.	Lung	Nerolidol inhibited alveolar-capillary barrier disruption and lung injury via the activation of the AMPK/Nrf-2/HO-1 signaling pathway.	(97)
LPS	Rat	Recombinant human EPO (rhEPO)	3000 IU Concomitant administration with LPS injection	N.A.	Kidney	rhEPO suppressed apoptosis via AMPK/SIRT1 pathway mediated autophagy.	(98)
LPS+CLP	Mouse	Resveratrol (polyphenolic compound)	50 mg/kg 4 and 16 h after LPS injection	Yes	Macrophages	Resveratrol inhibited the endotoxin tolerance in macrophages via CAMKKβ/AMPK activation	(99)
CLP	Rat	Salidroside (extract of plant Rhodiola rosea)	20 mg/kg 30 min before CLP surgery	N.A.	Lung /Macrophages	Salidroside reduced HMGB1 release through the AMPK/SirT1 signaling pathway and attenuated lung injury	(100)
LPS	Mouse	Smiglaside A (extract of Chinese herb)	10 mg/kg 3 consecutive days before LPS injection	Yes	Lung /Macrophages	Smiglaside A promoted macrophage M2 polarization through the activation of the AMPK/PPARγ signaling pathway and ameliorated lung injury	(101)
CLP	Mouse	Stearoyl lysophosphatidylcholine (sLPC)	20 mg/kg 1 h before and 11 h after CLP or 4 and 16 h after CLP surgery	N.A.	Macrophages	sLPC inhibits the release of HMGB1 through the G2A/CaMKKβ/AMPK pathway	(102)
CLP	Mouse	Trimetazidine (anti-ischemic agent)	20 mg/kg 7 consecutive days after CLP surgery	Yes	Heart	Trimetazidine improved cardiac dysfunction by promoting neutrophil recruitment in an AMPK/Nrf2 dependent manner	(103)
LPS	Mouse	U75302 (leukotriene B4 BLT1 antagonist)	1 mg/kg 1 h before LPS injection	Yes	Heart	U75302 suppressed cardiac apoptosis, inflammation, and mitochondrial impairment via AMPK activation	(104)

AMbPK, AMP-activated protein kinase; CLP, Cecal ligation and puncture; LPS, Lipopolysaccharides; AICAR, 5-aminoimidazole-4-carboxamide ribonucleoside.

N. A., Not assessed.

### Drawbacks of AMPK activation

While a substantial body of evidence highlights the beneficial effects of AMPK activation, data also suggest that inhibiting AMPK can reduce liver injury. The AMPK inhibitor compound C reduces hepatocyte apoptosis and mitigates liver damage by inhibiting the pro-apoptotic protein JNK in a model of LPS/D-galactosamine-induced fulminant hepatitis (105). Additionally, inhibiting AMPK improves LPS-induced liver damage by suppressing the ROS/NF-κB signaling pathway (106). The role of AMPK in myocardial ischemic injury remains debated, with conflicting views on whether it is beneficial or harmful (107). Furthermore, in certain tumor types or under conditions of limited nutrients or hypoxia, AMPK has been observed to promote tumor growth (13).

## Discussion

Although AMPK activation shows promise in the treatment of sepsis, several barriers remain prior to translation to bedside use above and beyond the absence of clinical trials supporting the efficacy of this approach. First, sepsis is a highly heterogeneous condition characterized by the presence of multiple suphenotypes, each with distinct pathophysiological mechanisms and clinical outcomes (108–116). Additionally, subtype strategies using clinical, biomarker and transcriptomic data do not identify comparable patient populations with sepsis (117), suggesting precision medicine approaches to identifying which patients might respond to AMPK-targeted therapies would be challenging. Next, the diversity of cell types involved in the septic response further complicates the potential usage of AMPK activation strategies due to potential of unwanted off-target effects. Additionally, immunometabolic paralysis – a state of metabolic dysfunction in immune cells – is another potential barrier to effective sepsis treatment with AMPK activation. While AMPK is a central regulator of cellular metabolism, its role in reversing immunometabolic paralysis in sepsis is not yet fully understood. Emerging evidence suggests that AMPK activation may influence immune cell function, but the effects appear to be context-dependent, requiring more precise targeting strategies. Future research should focus on elucidating the specific pathways through which AMPK modulates immune metabolism during sepsis and developing biomarkers to guide the use of AMPK-targeted therapies in overcoming immunometabolic paralysis (44).

Recent studies have identified itaconate, a metabolite derived from the TCA cycle, as a significant modulator of immune responses, contributing to disease tolerance in sepsis (118). Itaconate is mainly produced by activated macrophages through the enzyme immune-responsive gene 1 (IRG1) and inhibits ferroptosis of macrophages via Nrf2 pathways in sepsis-induced acute lung injury (119). As a central regulator of cellular energy homeostasis, AMPK interacts with multiple metabolic pathways, including those linked to the TCA cycle. This interaction suggests that AMPK and itaconate may work together to modulate immune responses and metabolic reprogramming in sepsis given the role of mitochondrial TCA cycle metabolites in physiology and disease (120). Additionally, AMPK may influence itaconate production by regulating IRG1 expression, as AMPK can modulate inflammatory responses through altering macrophage polarization (121). Given that itaconate has been demonstrated to regulate AMPK signaling in hepatocytes (122, 123), this suggests a complementary approach to direct AMPK activation in modulating metabolic and inflammatory responses in sepsis.

Ultimately, AMPK plays a crucial role in numerous biological processes that are relevant to sepsis pathogenesis, and numerous studies suggest that either AMPK activation or deficiency can influence susceptibility to sepsis in ways that vary by age or sex (124, 125). It is exciting to consider the possibility of AMPK as a potential therapeutic target in human sepsis given the numerous pre-clinical studies demonstrating a benefit of AMPK activation and associative studies suggesting a potential benefit of metformin in critical illness. However, there is currently insufficient human evidence to support using metformin or other AMPK activators to treat septic patients in the ICU. Ongoing and future clinical trials may clarify the role (if any) in activating AMPK in human sepsis while future research focused on tissue-specific and condition-specific AMPK activators will hopefully clarify mechanisms underlying potential efficacy.
